# The Effects of a Small Dose of Tannin Supplementation on In Vitro Fermentation Characteristics of Different Forages

**DOI:** 10.3390/ani15091269

**Published:** 2025-04-29

**Authors:** Sytske de Jong, Fabiellen C. Pereira, Alejandro R. Castillo, Wilbert F. Pellikaan, Pablo Gregorini

**Affiliations:** 1 Animal Nutrition Group, Department of Animal Sciences, Wageningen University and Research, 6708 Wageningen, The Netherlands; sytske.dejong@wur.nl (S.d.J.); wilbert.pellikaan@wur.nl (W.F.P.); 2 Department of Agricultural Sciences, Faculty of Agricultural and Life Sciences, Lincoln University, P.O. Box 85084, Lincoln 7647, New Zealand; 3 Division of Agricultural and Natural Resources, Cooperative Extension, University of California, Merced, CA 95340, USA; arcastillo@ucanr.edu

**Keywords:** pastoral livestock production systems, rumen fermentation, tannins

## Abstract

Pastoral systems in New Zealand are under societal pressure due to their increasing negative environmental impact in terms of greenhouse gas emissions. This study was conducted to investigate the effects of supplementing a mixture containing hydrolysable and condensed tannins on the in vitro fermentation characteristics and gas production of three different forages, *Lolium perenne*, *Medicago sativa*, and *Plantago lanceolata*. Three fermentation runs of 48 h were conducted using the ANKOM gas production technique, with each pertaining to a particular forage with or without (control) tannin. Tannins were added to the fermentable substrate (i.e., forage) at a level of 0.3%. For each run, rumen fluid was collected from two fistulated Holstein Friesian × Jersey cows. The ANKOM RF gas production modules were used to monitor gas pressure and temperature every 5 min. At the end of each run, the pH of the fluid was measured, gas vials were taken for methane (CH_4_) measurements and liquor samples were taken to measure volatile fatty acids (VFA) and NH_3_ concentrations. The addition of tannins reduced the fractional rate of gas production for alfalfa (*p* ≤ 0.04) but increased it for ryegrass and plantain. There was a tendency for reduced gas production for ryegrass when tannins were added (*p* = 0.10). There was also a tendency for CH_4_ production to reduce (*p* < 0.10) and N_2_O to increase (*p* = 0.10) when tannins were added. Iso-butyrate tended to be lower for ryegrass control than to ryegrass with tannins (*p* = 0.08). Valerate concentration was lower for plantain control than to plantain with tannins. No effects were detected for gas composition (*p* > 0.05) or VFA concentrations (*p* > 0.05) when fermenting alfalfa. Under the condition of this study, these results may suggest that low-level tannin addition to the diet may affect rumen-fermentation pattern with a potential reduction of CH_4_ production in Lolium perenne-based diets. Further research is required on the effect of low levels of tannin supplementation under ex vitro and in vivo conditions as tannin supplementation effects might be substrate-dependent.

## Simple Summary

Pastoral systems in New Zealand are under societal pressure due to their increasing negative environmental impact in terms of greenhouse gas emissions. An in vitro study simulating rumen fermentation was conducted to investigate the effects of adding tannins at low levels to a cow’s diet composed of three different forages—ryegrass, plantain, and alfalfa. Several parameters of rumen fermentation and final products were measured. The results suggest that low-level tannin addition to the diet may affect rumen-fermentation pattern with a potential reduction of methane (CH_4_) production on ryegrass-based diets. It also suggests that tannin supplementation effects might be substrate-dependent, as the effects differed with different forages.

## 1. Introduction

Pastoral systems in New Zealand are under societal pressure due to their increasing negative environmental impact in terms of greenhouse gas emissions and N use inefficiencies that lead to waterways eutrophication and consequently human health issues [1]. Dairy cattle contribute to 25.7% of the gross greenhouse gas (GHG) emissions in New Zealand, of which 22.4% is related to methane (CH_4_) production and 3.3% with nitrous oxide (N_2_O) production (New Zealand’s Greenhouse Gas Inventory, accessed on 1 December 2024). Both GHGs are the most influential anthropogenic GHGs. Methane accounts for 27.2 CO_2_-equivalents, whereas N_2_O is even stronger and accounts for 273 CO_2_-equivalents [1]. Therefore, strategies to reduce GHG emissions in the intensive pastoral dairy sector are needed.

Tannins are polyphenol compounds that are found in some plants. Based on their molecular structure, tannins are primarily categorized into two major groups: condensed (CT) and hydrolysable tannins (HT). Depending on the group, they interact with microbial populations and plant primary and secondary chemical compounds in the rumen through several processes; for example, antibiotic activities and biding to other plant secondary compounds [2,3]. Several studies have reported that, through those processes, tannins help improve N utilization and reduce enteric CH_4_ production in ruminants [4,5].

Tannins can bind to proteins and fiber in the rumen, thereby affecting rumen-fermentation patterns and reducing digestibility when fed at adequate levels [2]. Different effects were reported with different levels of tannins supplementation, such as reduced NH_3_ production, blood urea N, and N excretion in urine when steers were fed quebracho tannin extract at high levels up to 45 g/kg DM [6], and reduced in vitro CH_4_ and NH_3_ production when quebracho tannin extracts or chestnut tannin extracts were added up to 200 g/kg DM [7]. Reduced total branched-chain VFA concentration and reduced CP and NDF apparent digestibility were also observed when dairy cows were fed a mix of quebracho and chestnut tannin extract at 1.8% of diet DM [8].

Few studies have reported the effects of tannin supplementation at low doses. Marshall, Beck [9] reported a reduction in methanogenic bacteria in the rumen and reduced urinary N excretion in dairy heifers fed a mixture of CT and HT at 0.15% of daily DMI. Pérez-Ruchel, Britos [10] reported in vitro reduced NH_3_-N concentrations (8.24 to 6.72 mg/dL), a reduced butyric acid concentration (16.25 to 13.57 mol/100 mol), and reduced apparent digestibility of CP and NDF with CT and HT supplementation at 10 g/kg DM. Both studies suggest a potential for a low-dose mixture of tannins (quebracho and chestnut) supplementation to affect rumen-fermentation patterns and reduce negative environmental impact.

In New Zealand, grazing systems are traditionally based on perennial ryegrass-based pastures. However, interest has risen in incorporating other forages like alfalfa, plantain, and chicory into the diet. Diverse diets may improve animal performance while reducing the environmental impact of ruminants [11,12]. Moreover, different forages present different fermentation patterns. When chicory or plantain was incorporated into a typical New Zealand ryegrass-based diet or used as a basal diet, Garrett, Beck [13] found increased in vitro gas production and reduced NH_3_ concentrations; consequently, diets containing these alternative forages to perennial ryegrass may modulate the effect and effectivity of tannins on rumen-fermentation pattern.

Research reporting the effect of low doses of tannins has been performed with perennial ryegrass-based diets [9,10], but there is still a lack of research exploring the effect of tannin supplementation on fermentation patterns of legumes (e.g., alfalfa) and herbs (e.g., plantain). Therefore, we hypothesize that there should be an effect of tannins on the in vitro gas production of other forages, even at low doses. To test our hypothesis, the present study aimed to evaluate the effects of a low level of tannin addition on in vitro fermentation characteristics, using ryegrass, plantain, or alfalfa as substrate.

## 2. Materials and Methods

All procedures outlined here were approved by the Lincoln University Animal Ethics Committee (AEC 2024-38).

### 2.1. Forage Sampling and Tannin Supplement

Three forages were separately used as substrates: perennial ryegrass (*Lolium perenne*), plantain (*Plantago lanceolata*), and alfalfa (*Medicago sativa*). Forage samples (50 g each) were collected from pasture on 29 October 2024 (Agricom, Lincoln, New Zealand), freeze-dried, and ground through a 1 mm sieve (ZM200; Retsh GmbH, Newtown, PA, USA). All forages were analysed for (DM, OM, WCS, NDF, ADF, CP, Digestibility and ME) using near infrared spectroscopy (Model: FOSS NIRS Systems 5000, FOSS, DK-3400 Hilleroed Denmark) (Table 1). For the treatment, a commercial powdered mixture of CT and HT (ByPro, Silvafeed, Italy) was added at 0.3% DM.

### 2.2. In Vitro Fermentation and Treatments

Three individual fermentations of 48 h each were run using the ANKOM RF Gas Production Systems (ANKOM), with each pertaining to a particular forage with or without (control) tannin. This resulted in six different treatments: perennial ryegrass control (RC), perennial ryegrass with tannin (RT), plantain control (PC), plantain with tannin (PT), alfalfa control (AC), and alfalfa with tannin (AT). For each run, seven jars were used for control treatment and seven for experimental treatment.

At each fermentation run, around 500 mL of ruminal fluid was collected from two lactating rumen-cannulated cows (Holstein-Friesian × Jersey; 550 kg live weight) grazing a pasture consisting of perennial ryegrass (*Lolium perenne*) and white clover (*Trifolium repens*) at Ashley Dene Research and Development station (Lincoln University, Springston, New Zealand). Rumen fluid was collected right after morning milking by taking rumen digesta from random locations within the rumen and straining it through cheesecloth into a warmed (39.5 °C) thermos flask and purged with carbon dioxide (CO_2_) to maintain anaerobic conditions.

Rumen fluid was restrained through cheesecloth and subsampled (20 mL per jar) into fermentation jars containing 80 mL of buffer solution. The buffer solution was prepared according to the operating instructions of ANKOM (2018) and consisted of combining two warmed (39.5 °C) solutions: buffer A (KH_2_PO_4_ at 10 g/L, MgSO_4_·7 H_2_O at 0.5 g/L, NaCl at 0.5 g/L, CaCl_2_·2 H_2_O at 0.1 g/L, and reagent grade urea at 0.5 g/L) and buffer B (Na_2_CO_3_ at 15.0 g/L and Na_2_S·9 H_2_O at 1.0 g/L) at a 5:1 ratio, and adjusted to a pH of 6.8.

Each fermentation jar contained 1.00 g of DM of substrate (corrected for residual DM) weighted in two F57 ANKOM bags, as per the randomized treatment to jar allocation for each run. Throughout the loading of fermentation jars, the mixture of rumen fluid and buffer medium was maintained at 39.5 °C within a water bath and purged with CO_2_. The loaded ANKOM jars fitted with the ANKOM RF Gas Production System (ANKOM) were placed within an oscillating incubator set at 60 revolutions per minute (Minitron, INFORS HT) for 48 h. The ANKOM RF Gas Production System automatically records the gas pressure and temperature every 5 min over the 48-h period. At the termination of the 48-h gas production period, the pH of the fluid was measured using a benchtop pH meter (Orion 2-star, Thermo Scientific, Waltham, MA, USA).

### 2.3. Chemical Analysis

To quantify in vitro degradability, the F57 bags were drained from the jars, washed in cold tap water, dried at room temperature, and then dried at 105 °C in the oven for 4 h. Subsamples of the rumen fluid–buffer solution and substrate were collected within 2-mL Eppendorf tubes, to determine VFA and CH_4_ concentrations, and another subsample was acidified (10 μL of 99% H_2_SO_4_) to determine NH_3_. These samples were stored at −20 °C until analysis. Volatile fatty acid concentrations were determined by gas chromatography (Playne, 1985) using a gas chromatographer (GC-2010, Shimadzu, Kyoto, Japan) fitted with an SGE BP21 30 m × 530 µm × 1.0 µm bore capillary column. Methane content was determined by gas chromatography using a gas chromatographer (Model 8610C, SRI Instruments, Torrance, CA, USA) with automated Gilson GX-271 auto samples (Gilson Inc., Madison, MI, USA). Ammonia content was determined using a fully automated clinical chemistry analyzer (RX Daytona+, Randox Laboratories Ltd., London, UK).

### 2.4. Statistical Analysis

Statistical analysis was conducted using R (R Core Team v.09.10, 2024) following a two-group experimental design, considering jar as the experimental unit. Gas pressure data were converted to mL produced using the ideal gas law and Avogadro’s number. The normal distribution of the data was assessed using a Q-Q plot and the Shapiro–Wilk normality test. pH, total gas production (mL), degradability (%), CH_4_ (mL), N_2_O (%), CO_2_ (%), NH_3_ (mM) and VFA concentrations (mM) were analyzed using ANOVA (car package) [14].

Linear contrasts were generated using the emmeans package [15] to compare gas production within forage (control and with tannins). The Ørskov and McDonald [16] model was used to analyze gas production data,

$$p=b(1-e^{-ct})$$

where *b* is the theoretical asymptote of the gas curve, *c* is the fractional rate of gas production (%/h), and *p* the gas production after time *t*. The Ørskov and McDonald [16] model was fit using the *nlme* function [17].

Results are presented as means with standard errors. Significance was declared at *p* ≤ 0.05 and trends at 0.05 < *p* ≤ 0.10.

## 3. Results

The chemical composition of the three forages used as substrate for the in vitro runs is shown in Table 1.

**Figure 1:**
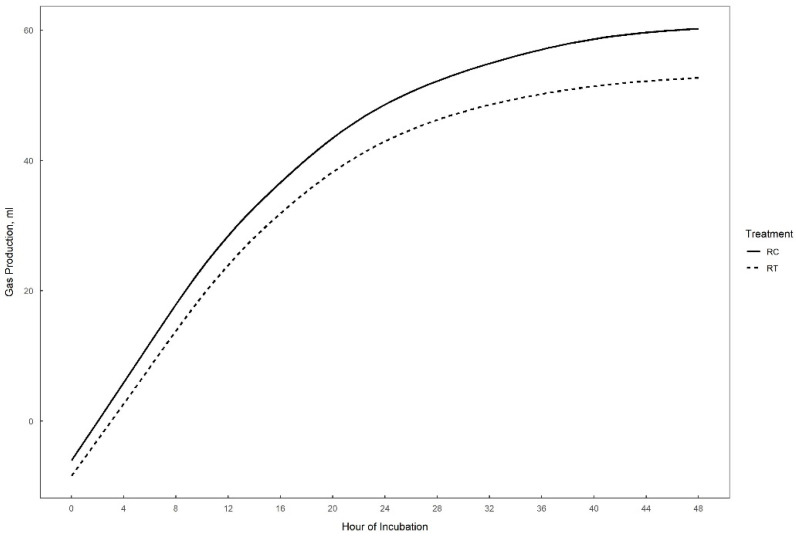
48 h in vitro gas production curve of perennial ryegrass with (RT) or without (RC) the addition of 0.3% tannins.

**Figure 2:**
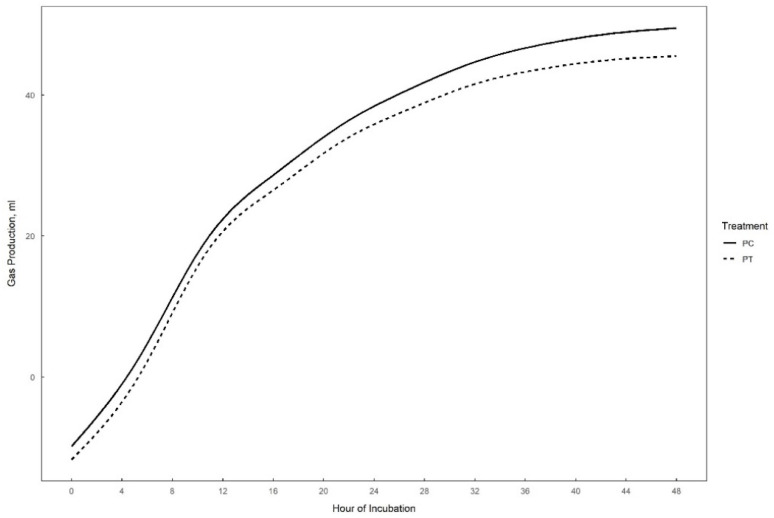
48 h in vitro gas production curve of plantain with (PT) or without (PC) the addition of 0.3% tannins.

**Figure 3:**
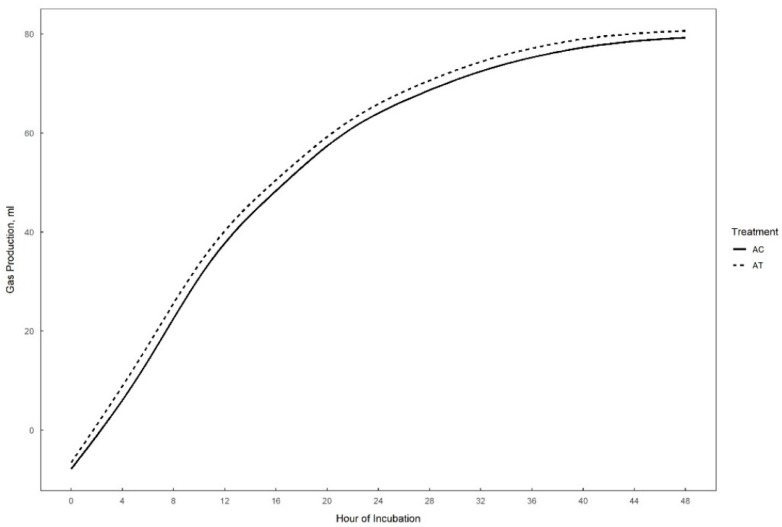
48 h in vitro gas production curve of alfalfa with (AT) or without (AC) the addition of 0.3% tannins.

Gas production over 48 h in vitro fermentation with or without supplementation of 0.3% tannins to the different substrates (perennial ryegrass, plantain, and alfalfa) is presented in Figure 1, Figure 2 and Figure 3. The theoretical asymptote of the gas production curve was not affected by the different treatments (*p* > 0.05). The fractional rate of gas production (%/h) was affected by treatments (*p* ≤ 0.04) (Table 2, Table 3 and Table 4). The fractional rate of gas production was greater for RC, PC, and AT than RT, PT, and AC, respectively. RC tended to have a greater total gas production (mL) than RT (*p* = 0.10) (Table 2).

Tannins did not affect perennial ryegrass degradability (%) (*p* = 0.20). CH_4_ (mL) production tended to be greater for RC than for RT (*p* < 0.10). N_2_O (%) tended to be lower for RC than for RT (*p* = 0.10). No effect of tannins was detected for CO_2_ (%) (*p* = 0.26), NH_3_ (mM) (*p* = 0.14) and VFA concentrations (mM). Only iso-butyrate tended to be lower for RC than RT (*p* = 0.08).

The inclusion of tannins did not affect the in vitro degradability or gas production of plantain (Table 3). In addition, no effects on VFA production were detected, except for valerate (mM) which was lower for PC as compared to PT (*p* = 0.05).

The only effect of tannins detected in alfalfa fermentation was the increased fractional rate of gas production (%/h) (*p* < 0.05).

## 4. Discussion

Tannins are polyphenolic plant secondary metabolites that can precipitate or crosslink the proteins, thus making them less prone to proteolysis; plus, tannins have the potential to reduce methane emissions in ruminants (Das et al., 2020). We hypothesize that low doses of tannin supplementation may alter the fermentation characteristics and products of different forages. Based on the results of the present study, we cautiously accept the general hypothesis, and we discuss our results within that premise. We first discuss the effects of tannins on in vitro fermentation characteristics of the forages tested in this study. Second, we provide a critical reflection on the method used for this experiment.

The fractional rate of gas production was greater for ryegrass, plantain, or alfalfa control or treatment (RC vs. RT, PC vs. PT, and lower for AC vs. AT). The reduction of this rate can be related to the effects of condensed tannins binding to fiber and protein, thereby reducing digestibility and gas production [2,3] and the antibiotic effect of hydrolysable tannins on the rumen microbiome [9,18]. Unexpectedly, AC had a slower fractional rate of gas production than AT, which contrasts previous findings reported on alfalfa fermented with quebracho or chestnut tannins [18]. There was a tendency (*p* = 0.1) for greater gas production (mL/g DM) at RC than at RT. This is supported by previous reports on in vitro fermentation adding 200 g/kg DM condensed or hydrolysable tannins to a total mixed ration mainly consisting of corn and timothy silage [7]. Again, tannins can bind to protein and fiber, reducing ruminal degradation and gas production [2,6]. However, the addition of tannins did not affect total gas production of ryegrass, probably due to the low addition level. This is supported by an in vitro study of Verma, Akpensuen [19] who reported reduced gas production in perennial ryegrass when tannin extracts containing both HT and CT were incubated with the substrate at 10–30 g/kg DM. Additionally, an in vitro study of Pérez-Ruchel, Britos [10] reported no differences in total gas production when adding a mix of quebracho and chestnut tannins at 10 g/kg DM to a diet mainly consisting of grass- and corn silage. Consequently, our study reinforces previous reports suggesting that tannins interact differently at different levels of addition with feed substrates. Its components and impact on rumen-fermentation dynamics seem to be mainly related to the level of addition.

The addition of tannins did not affect the degradability of any of the forage samples tested. This may be explained by our low level of tannin addition, which could have slowed down the fermentation rate without affecting the fermentation products such as degradability. Reduced in vivo ADMD was reported when using a mix of quebracho and chestnut tannins at 45–180 of DM [8]. Similar results were reported when using quebracho tannins at 30 and 45 g/kg DM [6], or supplementing chestnut tannins at 55 g/kg DM [20]. Others, investigating the effect of in vitro tannin supplementation, found that 10 g/kg DM of quebracho and chestnut tannins reduced ADMD [10]. Our study used a low supplementation level (3 g/kg DM), compared to the studies previously mentioned. Therefore, low tannin supplementation might affect fermentation rate, but not substrate degradability.

Methane production tended to be lower in RC than in RT. This could be related to a mild effect due to the low level of tannin addition–higher tannin doses would have resulted in clearer effects–on numbers of methanogens and fungi, as reported in an in vivo study feeding dairy heifers a corn-based diet [9] and an in vitro study on fermenting alfalfa [18]. Reduced numbers of methanogens (18.9–36.3%) and fungi (14.3–43.0%) were reported when chestnut tannins or a combination of tannins from chestnut and quebracho were added to alfalfa, which would potentially indicate reduced CH_4_ production [21]. Marked in vitro reductions of CH_4_ production (23.1–40.3%) were reported when adding quebracho or chestnut tannins at levels ≥ 100 g/kg DMI [7]. Therefore, a slight reduction trend in CH_4_ production, as the one detected in our study, might seem reasonable for ryegrass. Methane production was not affected by the addition of tannins when fermenting either plantain or alfalfa, with only numerical differences of similar magnitude as compared to RC vs. RT. We speculate that such a trend and the lack of effect on plantain and alfalfa are related to the type of study and inoculant, as we discuss below. Also, higher tannin levels. i.e., to explore dose-dependent responses. And address the potential microbial shifts leading to methane suppression (e.g., inhibition of methanogenic archaea and reduced hydrogen availability).

We detected only minor effects of tannins on rumen-fermentation pattern, in terms of VFA profile, with that being only detected in plantain and ryegrass. A reason for such an effect can be a shift in microbial populations [9,21], reducing the relative abundance of methanogens, bacteria, fungi and protozoa [21,22,23,24]. As shown here and in previous research, the type of tannin, supplementation level, and substrate can influence the effect on in vitro VFA production. Two studies investigated the effect of quebracho and chestnut tannins separately using the same substrate. Differences were detected in VFA production, indicating that the type of tannin influences fermentation patterns [7,18]. Additionally, other studies, using different supplementation levels of a mixture of quebracho and chestnut tannins (10 vs. 20 g/kg DMI), observed differences in the production of valerate and iso-butyrate [10,21], as well as butyrate (Zhuang et al., 2024). Our study found a trend for lower iso-butyrate production in RC vs. RT (*p* = 0.08) and lower valerate production for PC vs. PT. In contrast to our findings, in the previously mentioned study, valerate and iso-butyrate concentrations were reduced when supplementing tannins ≥ 20 g/kg DM, indicating that low levels of tannin addition as low as in our study could either have no or erratic effect on the production of this VFA.

The lower valerate production for PC vs. PT can be explained by a study investigating the effect of tannins, the same tannin supplement used in our study, on rumen microbiota. They found that tannins altered the rumen microbiome, particularly carbohydrate-degrading bacteria [25]. Valerate is produced as an end product of carbohydrate fermentation [26]. It was reported that tannins promoted Ruminococaceae bacteria, which are associated with greater valerate production [25,27]. Iso-butyrate is produced by the deamination of valine, leucine, and isoleucine [26]. It might be possible that tannin supplementation also promotes bacteria that are associated with iso-butyrate production, which might explain the finding for a tendency of lower iso-butyrate production for RC vs. RT.

### Increasing Publication Supporting the Null Hypothesis Would Help Researchers Save Unnecessary Work: Reflection on Method

In summary, the mild level of tannin effect on fermentation characteristics in this study can be attributed, firstly, to the purposely low level of tannin supplementation to avoid reduction in degradability, which would be negative to animal performance. The level of tannin supplementation in this experiment was based on two in vivo studies using a low level of the same commercial tannin mixture at 0.15 and 0.45% of DMI [8,9], in New Zealand and the USA. Both studies reported reduced urinary N, and methanogens, as well as greater molar proportion of propionate, without negatively affecting animal health in dairy cows. Of course, greater effects of tannins on rumen-fermentation pattern (e.g., reduction of CH_4_ and NH_3_ production) have been reported in vitro and are expected in vivo when using greater levels of tannin addition (1–10% of the diet) to the base diets; at the price of a reduction in ADMD and potentially negative consequences in the animal by reducing total DMI [7,10,18,28]. Secondly, the rumen fluid–inoculant–used in our study is the New Zealand standard for in vitro forage fermentations, i.e., originates from cows grazing perennial ryegrass and white clover. Therefore, the microbiota of such inoculant was only adapted to one of our tested forages; which helps to explain some of our results, though mild, more pronounced in ryegrass than in plantain and alfalfa (with no statistical effect but numerical differences supporting such trends) [21,29]. It is therefore suggested that, at the time of evaluating the effects of low levels of tannin supplementation on in vitro fermentation, the animal providing the rumen fluid should be adapted to the diet being fermented. Little information, however, exists on the adaptation period of rumen and animal metabolism to tannins ingestion. Further research is needed in this regard.

## 5. Conclusions

Adding low levels of a mixture containing condensed and hydrolysable tannins to different forages in vitro fermentation slightly alters fermentation patterns and gas composition, with a potential reduction of CH_4_ production without reducing forage degradability. The magnitude of in vitro fermentation effects of tannins might be substrate and supplementation level dependent. Further, ex vitro and in vivo research is needed in a range of low-dose tannin supplementation potential to mitigate methane emissions in grazing systems.

## Figures and Tables

**Table 1:** Chemical composition of the substrates—forages—(% of DM unless otherwise noted).

Item	Perennial Ryegrass	Plantain	Alfalfa
DM, % as fed	94.97	94.74	94.53
OM, % as fed	91.52	86.86	91.49
Ash % (DM)	8.8	15.2	8.7
WSC ^1^ % (DM)	22.67	14.13	13.54
NDF % (DM)	37.79	15.49	20.29
ADF % (DM)	19.30	16.84	18.25
CP % (DM)	18.24	13.34	29.68
Digestibility, % (DMD)	83.84	79.04	79.66
MJ, ME/kg DM	12.72	11.40	12.10
Tannin, % (DM) ^2^	-	0.5	-

^1^ WSC = water-soluble carbohydrates. ^2^ Taken from Stewart (1996) [2].

**Table 2:** In vitro gas production and fermentation characteristics for perennial ryegrass supplemented with (RT) or without (RC) 0.3% tannins after 48 h of incubation.

Variables	Treatment		*p*-Value
	RC	RT	
pH	6.05 ± 0.03	6.05 ± 0.05	0.93
Theoretical asymptote of the gas curve	71.7 ± 1.92	65.9 ± 3.271	0.21
Fractional rate of gas production (%/h)	0.0443 ± 0.00	0.039 ± 0.00	<0.0001
Total gas production (mL/g DM)	60.1 ± 3.03	52.6 ± 10.80	0.10
Degradability (%)	52.7 ± 2.21	51.3 ± 1.56	0.20
CH_4_ (mL/L)	3.52 ± 0.37	3.16 ± 0.34	0.05 < *p* < 0.10
N_2_O (%)	1.0 ± 0.64	2.0 ± 1.31	0.10
CO_2_ (%)	76.3 ± 10.50	70.0 ± 6.38	0.26
NH_3_ (mM/L)	29.5 ± 0.59	30.0 ± 0.50	0.14
Acetate (mM/L)	40.40 ± 2.61	38.00 ± 3.33	0.16
Propionate (mM/L)	18.84 ± 1.56	17.98 ± 1.79	0.36
Butyrate (mM/L)	9.98 ± 0.73	9.93 ± 1.01	0.92
Valerate (mM/L)	2.77 ± 0.17	2.64 ± 0.30	0.33
Iso-butyrate (mM/L)	1.30 ± 0.23	1.51 ± 0.18	0.08
Iso-valerate (mM/L)	1.99 ± 0.17	1.98 ± 0.22	0.87
Hexanoate (mM/L)	0.72 ± 0.08	0.69 ± 0.14	0.59

**Table 3:** In vitro gas production and fermentation characteristics for plantain supplemented with (PT) or without (PC) 0.3% tannins after 48 h of incubation.

Variables	Treatment		*p*-Value
	PC	PT	
pH	5.87 ± 0.03	5.89 ± 0.07	0.45
Theoretical asymptote of the gas curve	65.3 ± 2.20	62.1 ± 2.22	0.30
Fractional rate of gas production (%/h)	0.034 ± 0.00	0.032 ± 0.00	0.04
Total gas production (mL/g DM)	49.6 ± 4.42	45.5 ± 4.68	0.12
Degradability (%)	47.2 ± 2.76	47.0 ± 1.98	0.91
CH_4_ (mL)	2.84 ± 0.45	2.71 ± 0.27	0.50
N_2_O (%)	2.2 ± 1.95	2.0 ± 0.85	0.79
CO_2_ (%)	74.6 ± 12.8	71.0 ± 5.52	0.51
NH_3_ (mM/L)	21.6 ± 0.39	22.0 ± 0.63	0.21
Acetate (mM/L)	36.33 ± 2.04	36.54 ± 1.88	0.85
Propionate (mM/L)	16.33 ± 0.82	16.14 ± 1.00	0.70
Butyrate (mM/L)	7.27 ± 0.49	7.29 ± 0.29	0.94
Valerate (mM/L)	1.43 ± 0.09	1.53 ± 0.09	0.05
Iso-butyrate (mM/L)	0.90 ± 0.19	0.79 ± 0.10	0.18
Iso-valerate (mM/L)	1.16 ± 0.12	1.15 ± 0.08	0.86
Hexanoate (mM/L)	0.58 ± 0.08	0.61 ± 0.06	0.44

**Table 4:** In vitro gas production and fermentation characteristics for alfalfa supplemented with (AT) or without (AC) 0.3% tannins after 48 h of incubation.

Variables	Treatment		*p*-Value
	AC	AT	
pH	6.12 ± 0.04	6.12 ± 0.03	0.88
Theoretical asymptote of the gas curve	95.4 ± 1.79	94.2 ± 1.92	0.67
Fractional rate of gas production (%/h)	0.043 ± 0.00	0.046 ± 0.00	<0.0001
Total gas production (mL/g DM)	79.3 ± 2.90	80.5 ± 4.70	0.56
Degradability (%)	58.6 ± 1.29	58.2 ± 1.37	0.61
CH_4_ (mL)	5.97 ± 0.47	5.83 ± 0.95	0.72
N_2_O (%)	1.2 ± 0.71^2^	1.3 ± 0.37	0.72
CO_2_ (%)	74.7 ± 5.30	70.4 ± 11.70	0.41
NH_3_ (mM/L)	40.2 ± 0.60	39.9 ± 0.80	0.55
Acetate (mM/L)	43.09 ± 2.09	44.07 ± 1.49	0.34
Propionate (mM/L)	18.55 ± 1.10	18.99 ± 0.59	0.37
Butyrate (mM/L)	9.40 ± 0.50	9.56 ± 0.45	0.52
Valerate (mM/L)	2.56 ± 0.24	2.56 ± 0.18	1
Iso-butyrate (mM/L)	1.62 ± 0.15	1.61 ± 0.12	0.94
Iso-valerate (mM/L)	2.87 ± 0.22	2.95 ± 0.18	0.46
Hexanoate (mM/L)	0.69 ± 0.08	0.71 ± 0.04	0.42

## Data Availability

The raw data supporting the conclusions of this article will be made available by the authors on request.
